# Correlation Analysis between Landscape Metrics and Water Quality under Multiple Scales

**DOI:** 10.3390/ijerph15081606

**Published:** 2018-07-28

**Authors:** Xin Zhang, Yuqi Liu, Lin Zhou

**Affiliations:** 1 State Key Laboratory of Remote Sensing Science, Institute of Remote Sensing and Digital Earth, Chinese Academy of Sciences, Beijing 100101, China; liuyq2015@radi.ac.cn; 2 University of Chinese Academy of Sciences, Beijing 100049, China; 3 College of Remote Sensing Information Engineering, Wuhan University, Wuhan 430079, China; 2015302590149@whu.edu.cn

**Keywords:** non-point source pollution, watershed, multiscale, multiple linear regression, landscape metrics, water quality index

## Abstract

Non-point source pollution is the main factor causing water quality deterioration. Landscape patterns affect the transmission of non-point source pollutants. Many studies have been carried out to analyze the correlation between landscape patterns and water quality, while most former studies neglected the scale effect. The Jiulong River basin in southeast China was selected as the study area. Based on a landscape cover map generated from satellite images, we determined the riparian buffer zones with different widths, set the catchment as the complementary scale, and then established the multiple linear regression models to explore the relationship between landscape metrics and water quality indices at different scales. The degree of significance of the effect of various landscape metrics on the water quality at different scales was quantitatively analyzed in this paper by using multiple linear regression analysis. The results showed that not only the impact of landscape metrics but also the influence of land cover type on the water quality indices would vary when the spatial scale changed. The credible regression models established in this study can help regional managers understand the correlation between landscape and water quality, and the regression results can be used for land use allocation in a watershed.

## 1. Introduction

The 2016 environment bulletin issued by China’s State Environmental Protection Administration demonstrated that river sections with water quality grades of I–III accounted for 67.8% among 1940 national control sections, while a water quality grade of IV accounted for 32.2%; the major pollution indices were pH, dissolved oxygen (DO), chemical oxygen demand (COD_Mn_), total phosphorus (TP), ammonia nitrogen amount (NH3-N), and total nitrogen (TN). Both point source pollution and non-point source pollution affect water quality. While point source pollution has been fairly controlled, non-point source pollution is extensive and difficult to quantify [1]. Hence, non-point source pollution has been the main factor of water pollution during urbanization [2]. Agricultural development has also aggravated the extent of non-point source pollution. Based on the premise that agricultural development will continue, examining the relationship between source-sink landscape patterns and non-point source pollution has become a major aspect of controlling non-point source pollution [3].

The spatial distribution of the landscape pattern in a watershed is linked with the process of non-point source pollution. Some landscapes, such as cultivated land and residential land, contribute to the non-point source pollutants; some landscapes can inhibit or absorb the pollutants, including forestland and unused land [4]. Thus, changes in landscape patterns will have a significant effect on the water quality. Landscape metrics can characterize the spatial structure of landscape patterns. In order to examine the relationship between landscape pattern and water quality indices, the correlation analysis method has been utilized.

Remote sensing (RS) data have become the significant data source of landscape pattern research, as they are multitemporal, multiresolution, and synchronous observations [5]. Jha et al. provided the perspective that the technology of land and water management developed promptly following the development of remote sensing and geographic information system (GIS) techniques [6]. This paper also mentioned that RS and GIS techniques would contribute greatly to the following six aspects: the exploration and evaluation of groundwater resources, the selection of irrigation areas, the simulation of groundwater flow and pollution based on GIS, the assessment of groundwater pollution and protection of groundwater, the estimation of natural irrigation areas, and the analysis of hydrogeological data.

Many studies have been carried out on examining the relationship between landscape patterns and water quality metrics. Varanka et al. studied the impact of geomorphological factors on river water quality (at the catchment scale, as well as water quality indices including TP, TN, pH, and water color; geomorphological factors covered variables regarding topography, bedrock, and surface ground material; and the Spearman’s rank correlation test was applied to study the correlations among variables [7]. Ou et al. analyzed the correlation between landscape pattern and non-point source pollution and studied the tendency for spatiotemporal change in landscape metrics to reflect the response of natural land cover on agricultural development [8]. The results of the landscape metrics reflected that the influence of agricultural development on the regional ecosystem changed at different time. Li et al. studied the relationship between landscape characteristics and water quality and analyzed the effect of various landscapes on water quality by using the Pearson’s correlation coefficient method, and the relationship between landscape features and water quality in each season was analyzed using the stepwise multiple regression model [9]. The results showed that the composition and spatial structure of the landscape greatly affected water quality. Huang took the relationship between land use distribution and water quality into account and found that most water quality indices were negatively correlated with the proportion of urban area and was positively correlated with the forest area [10]. Shen et al. analyzed the quantified relationship between landscape indices and water quality at the landscape scale. The results showed that the effect of the landscape pattern on water quality could be characterized by the patch density of a water body, the largest patch index of a forest, and the proportion of land usage classes [11]. Li et al. evaluated the impact of urbanization development on water quality and studied the relationship between landscape patterns and water quality indices [12]. Clear differences existed in the results due to landscape diversity and differences in the transmission process of non-point source pollutants. Gonzales-Inca studied the linkages between 21 years of water quality data from 16 agricultural watersheds and landscape metrics by using a generalized linear model and multivariate redundancy analysis [13]. The landscape indices were derived at three functional scales: watershed-wide, saturation-excess zone and riparian zone. In addition, the author mentioned that the vegetation index was the significant indicator of nitrate content in autumn. Oliverira et al. analyzed the linkage between landscape pattern and five selected water quality indices using different zones of analysis: riparian buffers and exclusive contribution areas. It is worth noting that a Land Cover Pollution Index (LCPI) was proposed in the study to replace the single land use types and study the relationship between landscape pattern and water quality [14]. The results demonstrated that LCPI could explain the linkage between landscape pattern and water quality more effectively than the individual land use category, and the phenomenon was more distinguishable at the riparian buffer scale. Xiao et al. proposed a novel method to quantify the impact of human activities in rural areas on soil resources and evaluated the relationship between five landscape metrics and three human activity indicators [15]. The results of the analysis indicated the higher degree of landscape fragmentation during the study years. Su quantified the agricultural landscape pattern changes in response to urbanization at the ecoregional scale by integrating RS, GIS, landscape metrics analysis, and spatial regression [16]. The results showed that the urbanization process could be characterized by four indices: gross domestic product (GDP), total population, non-agricultural population proportion (NAPP), and the expansion intensity index (EII). In addition, EII was the most effective urbanization indicator explaining agricultural landscape pattern changes at the ecoregional scale. Shi researched the changes in water quality indices under different land use types by applying redundancy analysis [17]. The results reflected a more distinguishing impact of landscape patterns on water quality and a stronger contribution of land use patterns on the water quality at the riparian scale than at the catchment scale. Lv et al. characterized the landscape patterns by using proportions of land use and five land scape metrics and analyzed the correlation between landscape characteristics and water quality parameters at different buffer zones, ranging from 200 to 1500 m. The results indicated that the relationship between landscape pattern and water quality was scale-dependent [18]. Xiao et al. found that both scales and seasonality play important role when analyzing the relationships between landscape characteristics of different land use types [19]. However, the metrics describing landscape composition and pattern included the percentage of farmland (%FA), orchard (%OR), forest (%FO), built-ups (%BU), and water (%WA), and the four spatial scales were 100 m site buffers, 500 m site buffers, 1000 m site buffers, and 2000 m site buffers [19].

By analyzing the related studies, we noted that the traditional studies usually selected riparian buffers with different widths as the study scales and evaluated the linkage between landscape patterns and water quality by comparing the relationships established at various buffer zones. The drawback of this method is that the riparian buffer zones with different widths cannot reflect the scale effect of a given relationship. Dividing the buffer zones artificially destroys the original integrity of the landscape, and it is still debatable whether the landscape region after buffer zone division can be regarded as an independent study scale. So, the transmission of pollutants along with surface runoff in a catchment can be regarded as an independent ecological process. Thus, a new spatial scale is needed as the complement of riparian buffers. In this paper, the river catchment was selected as the complementary scale, in addition to the traditional riparian buffer zones. A novel method for analyzing the correlation between multiscale landscape patterns and water quality indices based on RS techniques was proposed in this paper to solve the uncertainty problem of the appropriate scale when studying the linkage between landscape patterns and water quality. Traditional landscape metrics, such as the number of patches (NP), the patch density (PD), the largest patch index (LPI), and the landscape shape index (LSI), were usually used in the related studies to characterize the spatial structures of landscape patches in the study area [19]. The minimum hydrological response unit with a single land use and soil type is the smallest study unit of the hydrological response unit landscape contrast index (HRULCI) [20]. HRULCI can reflect the different effects of various landscapes on the transmission of non-point source pollutants. As a different index, HRULCI was computed to analyze the correlation between landscape metrics and water quality under multiple scales in this paper.

## 2. Study Areas and Data Sources 

Jiulong River is the second-largest river in Fujian Province, with a total length of 1285 km. The North, South, and West Rivers are main streams in the Jiulong River basin [21]. The watershed covers Longyan, Longhai, Zhangping, and other districts, which are bounded between 116°47′ E to 118°02′ E and 24°13′ N to 25°51′ N [4]. The length of Jiulong River is about 258 km, the drainage area is about 14,741 m^2^, and the average sediment discharge is 2 million 461 thousand tons. The main streams include the North stream, the South stream, and the West stream. Jiulong River is an important source of drinking water and agricultural irrigation water in the study area. The health of a water body guarantees human productivity and the development of agriculture. The watershed is in a subtropical marine monsoon climate. The precipitation in this basin is abundant, and the amount of rainy days per year ranges from 100 to 200. In addition to agricultural production activities, the causes of pollution in the basin include livestock pollution, water resource overexploitation, industrial pollution, and the delayed sewage disposal.

**Figure 1:**
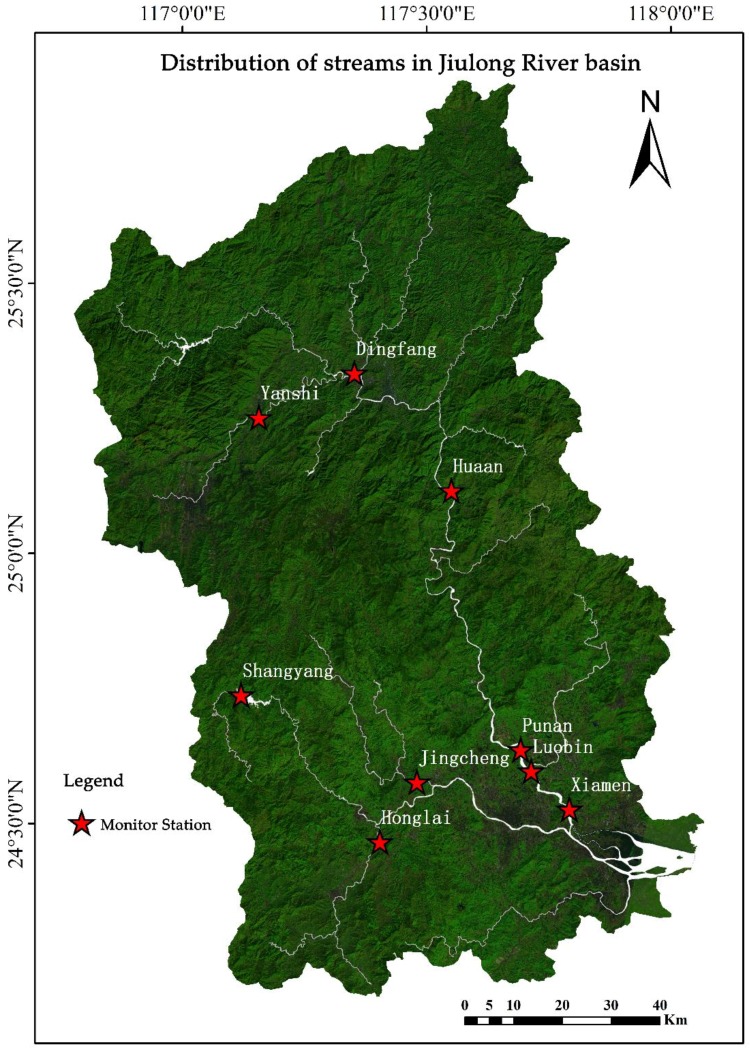
Monitoring sites in Jiulong River basin.

Landsat OLI images of the Jiulong River basin in 2017 were selected as the data source for landscape interpretation [22]. The digital elevation model (DEM) data with the resolution of 30 m were acquired from ASTER GDEM which was jointly launched by The Ministry of Economy, Trade and Industry of Japan and the United States National Aeronautics and Space Administration (NASA). Soil type datasets were provided by the Institute of Soil Science for the Second National Land Investigation, and the annual precipitation data were obtained from the Nation Meteorological Data Sharing Platform. The consumption data of fertilizers were acquired from statistical yearbooks for Fujian Province. The hydrological monitoring data came from the weekly report of water quality on the official website of the Fujian Provincial Department of Environment Protection, and the monitoring data were processed to obtain the annual hydrological data of different monitoring sites in the study year. The monitoring indices of water quality include pH, DO, COD_Mn_, TP, NH3-N, and TN. The spatial distribution of the main monitoring sites in the Jiulong River basin is displayed in Figure 1.

The annual water quality indicators data of major sites in 2017 basin are showed in Table 1.

Aiming to validate the accuracy of the multiple linear regression model established to study the relation between multiscale landscape metrics and water quality indices, the monitoring data of Huaan Xipo and Xiamen Jiangdong in 2017 were applied as the verification data for this paper. We proved the equation accuracy by comparing the simulated data with the actual monitoring data in this study.

## 3. Methods

### 3.1. Division of Multiscale Riparian Buffers and Calculation of Indices

**Figure 2:**
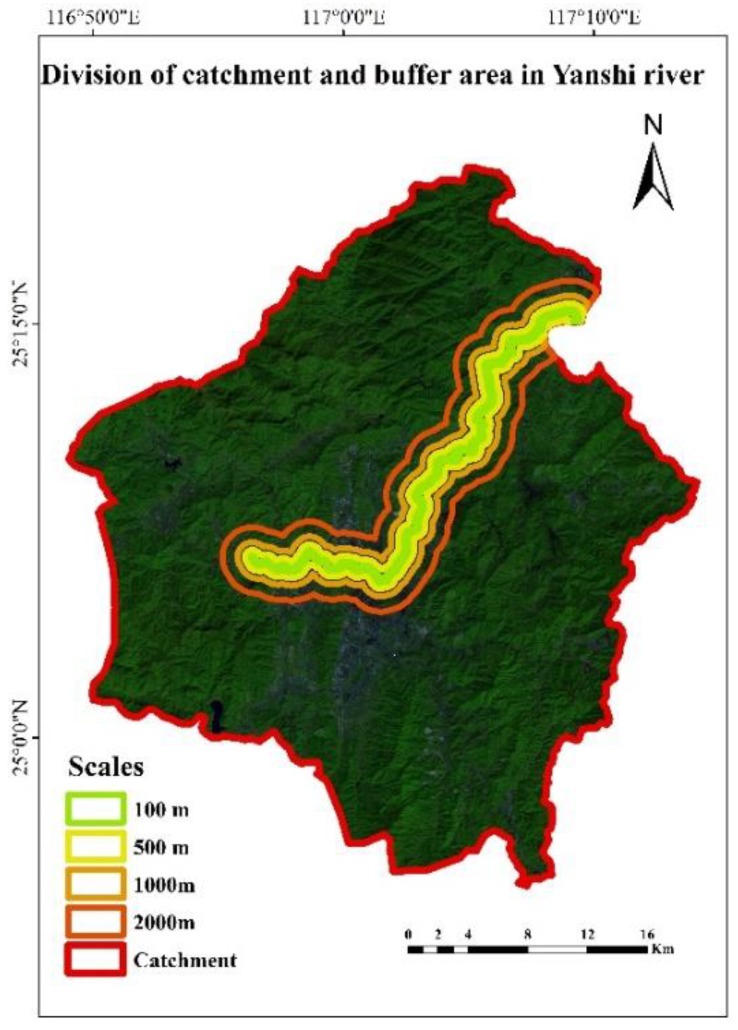
The division of buffer zones in Yanshi River.

The water system results of the Jiulong River basin in 2005, 2010, 2014, and 2017 were obtained on the basis of DEM data and the extraction results of the water. The distance from a certain landscape to the water has a great impact on the transferring process of non-point source pollutants along with the surface runoff with the precipitation process. Compared with the landscape that is farther away, the intensity of the landscape near the body of water is stronger. The transmission of non-point source pollutants in the watershed is a spatiotemporal process; thus, it is crucial to select the study scale when analyzing the relationship between landscape patterns and water quality. According to the former studies [23,24], we established riparian buffer zones with distances of 100 m, 500 m, 1000 m, and 2000 m. The buffer zones established in the Yanshi River sub-watershed are showed in Figure 2. Yanshi River is a tributary of the North River. It should be noted that the selected channel is a first-order stream.

Eight landscape metrics including the number of patches (NP), the patch density (PD), the largest patch index (LPI), the landscape shape index (LSI), the area weighted mean shape (AWMSI), the mean nearest neighbor distance (ENN_MN), the interspersion and juxtaposition index (IJI), and the aggregation index (AI) were computed by the FRAGSTATS platform which is a computer software program produced by Kevin McGarigal, SA Cushman and E Ene at the University of Massachusetts, Amherst [25]. Hydrological Response Unit Landscape Contrast Index (HRULCI) was computed according to the specific steps introduced in a former study [20]. Table 2 contains specific information on the 9 metrics [17,26,27].

The calculation results of the landscape metrics in the Yanshi River buffer zones in 2017 are displayed in Table 3. NP reflects the amount of patches in the certain area, and NP increased with the increase of the width of buffer zones. The decrease of PD indicated that the extent of fragmentation in 100 m, 500 m, 1000 m, and 2000 m also gradually decreased. LSI, indicating the overall complexity, increased when the width rise from 100 m to 1000 m.

### 3.2. Landscape Metrics Calculation at the Catchment Scale

The transmission of pollutants along with surface runoff in a catchment can be regarded as an independent ecological process. The river catchment was selected as the complementary scale in addition to the traditional riparian buffer zones. The division results of the catchment in the Yanshi River sub-watershed are showed in Figure 2. 

The calculation results of the landscape metrics in the Huaan Xipo catchment area in 2005, 2010, and 2014 are displayed in Table 4.

### 3.3. Analysis of the Relation between Spatiotemporal Landscape Metrics and Water Quality Indices

In this paper, all the water quality monitoring data were collected from the national water quality monitoring station in China, and the sampling frequency was once a week. Analysis of the linear or non-linear relationship between landscape metrics and water quality indices were performed in existing studies. The conclusions obtained from such studies are not comprehensive because the specific ecological process has not been taken into account and the scale effect has been neglected. Combined with non-point source pollution and the transmission of pollutants, the relationship between landscape metrics and water quality was studied by using multiple linear regression analysis at multiple scales [28]. The steps of the multiple linear regression model include the determination of the explanatory variables and explained variables, the determination of the regression model, the establishment of the regression equation, the verification of the equation, and the generation of a prediction using a regression equation.

## 4. Results and Discussion

We established the multiple linear regression equation between the landscape metrics and water quality indices at the scale of buffer zone and catchment with the support of SPSS.

### 4.1. Linear Analysis of pH and Landscape Metrics

The linear regression models simulated by regression analysis are shown in Table 5. The influences of landscape metrics on pH are different at various buffer zones and catchments. According to the criterion that two variables have a linear correlation only when significance is less than 0.05, the relationship between pH and landscape metrics calculated in this paper was non-linear. Meanwhile, it is notable that the significance (Sig) of the established models varied with the change in the spatial scales. The value of Sig improved promptly when the buffer width increased from 100 m to 500 m, and Sig remained stable after this great increase. Hence, it is inferred that the landscape metrics at a smaller scale have greater explanatory capacity for water quality indices in this paper.

According to the further analysis of the significance of pH and different landscape metrics at the 100 m buffer zones (Table 6), NP and LSI have great impact on pH, indicating that the fragmentation degree and overall shape complexity of landscapes are of significance for pH.

### 4.2. Linear Analysis of DO and Landscape Metrics

On the basis of the Sig values in Table 7, DO and landscape metrics did not show a great linear correlation in this paper, whereas with the increase in spatial scales, Sig dropped with the raise in the study scale, indicating that correlation models established at a large scale have better explanatory power than those at a small scale. The values of Sig at the 2000 m buffer and river catchment did not show a decreasing tendency, and we speculated that the reason lies in the small distances between monitoring sites in the Jiulong River. The catchments divided in this study may overlap with the 2000 m buffer zones, affecting the significant results of the relational models. Sig was the lowest at the 1000 m scale, but the model coefficient table indicated that there were no landscape metrics that related linearly with DO. We concluded that DO had a non-linear relation with landscape metrics.

### 4.3. Linear Analysis of COD_Mn_ and Landscape Metrics 

COD_Mn_ had a clear linear correlation with the landscape metrics at the catchment scale in the Jiulong River basin on the basis of the significance values in Table 8. The Sig of the linear regression models that was established for the COD_Mn_ and landscape metrics decreased rapidly with the improvement in the spatial scale. The Sig values of the models at the riparian buffers were all larger than 0.05, demonstrating that COD_Mn_ had no linear correlation with the landscape metrics at the buffer scales, while Sig at the catchment scale was 0.029, and thus, they had a significant linear relationship. The reason for this phenomenon was that compared with the riparian buffers, the influence of landscape pattern on water quality at the catchment scale was more suitable for the transmission of non-point source pollutants (which mainly refer to the oxidizable substances) in a watershed. The transmission of oxidizable substances along with surface runoff is a spatiotemporal process, and the division of the riparian buffers cuts apart the continuous space-time process. Hence, the linear regression model established at the catchment scale is the most convincing in the study of the relationship between landscape metrics and COD_Mn_.

The regression coefficients at the catchment scale are showed in Table 9. ENN_MN had the greatest impact on COD_Mn_ according to the results of significance. Furthermore, HRULCI significantly influenced COD_Mn_ because HRULCI was calculated based on the minimum hydrological unit, and the catchment consisted of several minimum hydrologic response units.

The linear regression model of the catchment scale calculated according to Table 9 is shown below.

(1) $$\mathrm{CODMn}=-7.8802\times 10^{-7}\times \mathrm{NP}+0.31\times \mathrm{PD}-0.009\times \mathrm{LPI}-0.01\times \mathrm{LSI}+0.024\times \;\mathrm{AWMSI}-0.017\times {\mathrm{ENN}}_{\mathrm{MN}}-0.063\times \mathrm{IJI}+0.207\times \mathrm{AI}+11.483\times \mathrm{HRULCI}-15.520,$$

### 4.4. Linear Analysis of TP and Landscape Metrics

The TP and landscape metrics were more closely related compared with other water quality indices based on Table 10. The credible linear regression model was established at every riparian buffer, and spatial scales had a slight effect on TP. F was lowest at the 500 m buffer scale, and values in the rest of the buffer scales were almost the same. In contrast, the significance value at the catchment scale was much higher, and the occurrence of this result was inevitable, which did not indicate that the study at the catchment was meaningless. The residential land and cultivated land are mainly distributed on both sides of the river, and non-point source pollutants consisting of phosphorus enter the water body and have a more significant influence on water quality than those at farther landscapes. Compared with the river catchment in which forestland inhibits the non-point source pollution, landscape metrics have greater effects on water quality indices at the riparian buffers. 

Although the linear regression models of TP and landscape metrics established at different buffer scales all had high accuracy, TP correlated closely with different metrics at different scales. According to the regression coefficients calculated at different buffer scales, TP had a linear relation with PD, LPI, ENN_MN, IJI, and AI at the 100 m scale and correlated with PD at the 500 m scale. TP had a significant correlation with LPI at the 1000 m scale and related linearly with LPI and AWMSI at the 2000 m buffer. This phenomenon indicated the importance of scale in the study of the source-sink landscape pattern. The significance of the regression model and the effect of landscape metrics on water quality indicators will change at different study scales. It was proven that the appropriate scale for analyzing the relationship between TP and landscape indices in the Jiulong River basin was the 500 m riparian buffer zone.

The linear regression model established according to Table 11 at the 500 m buffer was showed as follows.

(2) $$\mathrm{TP}=-4.916\times 10^{-6}\times \mathrm{NP}+0.01\times \mathrm{PD}+0.02\times \mathrm{LPI}+0.002\times \mathrm{LSI}-0.043\times \mathrm{AWMSI}+0.001\times \;{\mathrm{ENN}}_{\mathrm{MN}}+0.001\times \mathrm{IJI}+0.008\times \mathrm{AI}-0.213\times \mathrm{HRULCI}-0.848,$$

### 4.5. Linear Analysis of NH_3_-N and Landscape Metrics

NH3-N did not show a salient linear correlation with landscape metrics by analyzing the significance values in this study in Table 12. Significance values of the regression models at the 500 m buffer and the 2000 m buffer were approximately 0.07 and close to 0.05; thus, NH3-N and landscape metrics had weak correlations at the two scales. Based on further analysis of the significance between NH3-N and different landscape metrics at the two scales, it was inferred that NH3-N was weakly related to PD at the buffer zones with a width of 500 m, and NH3-N had a salient correlation with LPI and AWMSI, while it was weakly correlated with IJI and HRULCI at the 2000 m scale. Although the significance values of the linear regression models changed slightly at different study scales, the relationship between NH3-N and various landscape metrics changed promptly, indicating that the research scale was of great significance. The appropriate scale should be selected according to the specific ecological process.

The linear regression model of NH3-N and landscape metrics at the 2000 m scale was calculated on the basis of Table 13, which was shown as follows.

(3) $$\begin{array}{l} \mathrm{NH}3N=-3.974\times 10^{\;-5}\times \mathrm{NP}+0.051\times \mathrm{PD}+0.03\times \mathrm{LPI}+0.022\times \mathrm{LSI}-0.243\times \\ \mathrm{AWMSI}-0.003\times {\mathrm{ENN}}_{\mathrm{MN}}-0.04\times \mathrm{IJI}+0.067\times \mathrm{AI}-4.157\times \mathrm{HRULCI}+0.718, \end{array}$$

### 4.6. Linear Analysis of TN and Landscape Metrics 

The Sig of the regression models at different scales are showed shown in Table 14. TN was not correlated linearly with landscape metrics in this study. Significance dropped with the increase of study scale, indicating that landscape metrics had better explanatory power at the larger spatial scale. The model significance at the catchment scale was the lowest, which was similar to the conclusion of the analysis on COD_Mn_ analysis, and it was proven that the catchment scale was another crucial scale while studying the relationship between landscape patterns and water quality indices. Analyzing the regression significance of TN and various metrics at the catchment scale, ENN_MN had the most significant effect on TN compared with other landscape metrics. 

### 4.7. Accuracy Verification 

According to the landscape metrics shown by Huaan Xipo and Xiamen Jiangdong in 2017 and the multiple linear regression models of different metrics for water quality indices, we verified the accuracy of three regression models by examining the data of two monitoring sites (Huaan Xipo, Xiamen Jiangdong) in 2017. The simulated water quality indices are showed in Table 15.

According to Table 15, the linear regression equations for COD_Mn_, TP and NH3-N satisfied the verification data in Huaan Xipo. The simulated result of TP was in the hydrological monitoring data range, indicating that the regression model was credible in the Xiamen Jiangdong monitoring site. The regression results of COD_Mn_ are close to the range, but the result of NH3-N was far from the appropriate range. Considering the loss of monitoring data of Xiamen Jiangdong in 2017 and the credible simulation model of COD_Mn_ in Huaan Xipo, we speculated that this model was effective in Xianmen Jiangdong as well. The simulation of NH3-N at the Xianmen Jiangdong site was quite different from the monitoring data, which demonstrated that the regression model was not applicable at this site.

### 4.8. Discussion 

Landscape metrics have some indicative value for water quality, but their responses to varying scale is different [27]. Scale effect has been an important topic for a long time in the study of landscape patterns, and the determination of the appropriate scale is a crucial issue [20]. Sun et al. pointed out that landscape metrics were the dominant factors of water quality in the whole watershed and 200 m buffer zones and the impact of landscape metrics on water quality enhanced with the expansion of buffer zones [24]. Xiao et al. applied metrics describing landscape composition and pattern at class level and studied the correlation between landscape metrics and water quality parameters at 100 m site buffers, 500 m site buffers, 1000 m buffers, and 2000 m site buffers, and it was pointed out that scales played an important role when analyzing the relationship between landscape characteristics of different land use types and water quality [19]. Li et al. demonstrated the impacts of land use in riparian zone on water quality [29]. However, the transmission of pollutants along with surface runoff in a catchment should be further studied as an independent ecological process. We analyzed the correlation between landscape metrics and water quality parameters at different buffer scales and at the catchment scale in this paper. Some water quality indicators were closely correlated with landscape metrics at the buffer scales, and take TP as an instance, Sig of different buffer scales were lower than 0.05 which indicated that TP was correlated with landscape metrics at the buffer scales, however Sig at the catchment was larger than 0.05 and TP was not correlated with landscape metrics at the catchment scale. While for COD_Mn_, the results were different apparently. COD_Mn_ was correlated with landscape metrics at the catchment scale and the correlation was not significant at the different buffers.

The linear regression models constructed in this study can be divided into two categories. Dependent variables of the first class are TP, COD_Mn_, and NH3-N, and those of the second class are pH, DO, and TN. The first class models reflect the salient correlation between water quality indices and landscape metrics. In contrast, regression models of the second class indicate that the correlation between the corresponding water quality indicators and landscape metrics is not significant, and at a certain scale, such as pH and landscape metrics at the buffers and the catchment scale, is not correlated. We found that the significance of the regression model changed with the varied spatial scales by comparing the models of certain water quality indices established at different scales. The appropriate scale making the second kind of models incredible was not determined in this paper, but the tendency was such that the significance changed when different spatial scales were explained by analyzing the varied significance values. Taking pH, for instance, the results showed that the regression model at the 100 m scale was more credible than that at the large scale.

## 5. Conclusions

In the study on the relationship between landscape patterns and water quality indices in the Jiulong River basin, the linear regression equations of water quality indicators and landscape metrics were established at different scales in this paper. The catchment with ecological significance was employed as the complementary scale for the riparian buffer zones. The calculation results showed that the great advantage of the regression model of COD_Mn_ and landscape metrics was established at the catchment scale. It was proven that the relationship between the water quality indices and landscape metrics had a salient scale effect by comparing the multiple linear regression models at different scales. Not only the impact of landscape metrics on water quality but also the landscape categories with great influence on the water quality indices will change when the spatial scale changes.

The research results deepen the understanding of the relationship between landscape pattern and water quality in multi time-space scale and have significance for non-point source pollution control, such as the optimization of landscape spatial structure. Taking pH and DO as an example, this paper put forward a suitable scale for the relationship research between landscape pattern and the above water quality indicators. Through the analysis of the regression model of COD_Mn_ and landscape pattern index, we demonstrated that the catchment area is an important study scale to study the relationship between the landscape pattern and the water quality index in the basin. The above conclusions are of great significance to the research of the relationship between non-point source pollution and landscape pattern.

## Figures and Tables

**Table 1:** Annual water quality indicators dataset.

Monitoring Sites	pH	DO (mg/L)	COD_Mn_ (mg/L)	TP (mg/L)	NH_3_-N (mg/L)	TN (mg/L)
Honglai	7.133	8.280	2.730	0.153	0.400	11.490
Jingcheng	7.013	6.429	2.830	0.117	0.228	5.607
Dingfang	7.005	5.943	2.746	0.228	1.297	3.888
Punan	6.828	7.652	2.283	0.096	0.327	3.017
Shangyang	6.965	7.029	2.822	0.607	1.025	3.129
Yanshi	7.138	7.327	3.022	0.240	0.750	3.255
Luobin	6.992	6.706	3.490	0.136	0.331	5.324
Huaan	6.666	6.919	1.873	0.120	0.524	4.337
Xiamen	6.438	7.554	3.822	0.076	0.318	2.512
StdDev.	0.216	0.661	0.546	0.154	0.349	2.588

DO: dissolved oxygen; COD_Mn_: chemical oxygen demand; TP: total phosphorus; TN: total nitrogen.

**Table 2:** Introduction of landscape metrics.

Landscape Metrics	Description
Largest Patch Index (LPI)	LPI is the portion of the landscape that is occupied by the largest patch of the landscape.
Landscape Shape Index (LSI)	The sum of all patch perimeters is divided by an amount equivalent to the perimeter of a circle with the same area as the landscape area to calculate LSI.
Mean Nearest Neighbor Distance (ENN_MN)	ENN_MN is calculated only if at least two patches of a corresponding type occur. ENN_MN is the averaged distance from one patch to the nearest patch of the same landscape type. ENN_MN characterizes the landscape partially.
Interspersion and Juxtaposition Index (IJI)	IJI is calculated from the relationship between the length of each edge type and total edge of the landscape, divided by a term based on the number of landscape types.
Area Weighted Mean Shape Index (AWMSI)	AWMSI is computed by weighting patches according to their size.
Number of Patches (NP)	NP is the number of patches in a certain landscape type.
Patch Density (PD)	PD indicates the amount of patches per unit area in the landscape.
Aggregation Index (AI)	AI indicates the degree of patch clustering, ranging from 0 to 100.
Hydrological Response Unit Landscape Contrast Index (HRULCI)	HRULCI indicates the effect of a source-sink landscape on the transmission of non-point source pollutants from generating plots to a water body a) was calculated in this paper.

**Table 3:** Calculation results of landscape pattern index in different buffer zones.

Buffer Zone/m	NP	PD	LPI	LSI	AWMSI	ENN_MN	IJI	AI	HRULCI
100	503	50.111	13.852	23.599	3.864	86.429	42.285	67.751	0.997
500	1262	30.235	20.137	24.703	5.057	89.030	53.493	80.521	0.940
1000	2334	28.936	23.196	29.508	6.457	89.865	58.707	82.257	0.892
2000	5007	31.457	18.133	40.872	7.554	88.146	63.518	81.720	0.873

NP: Number of Patches; PD: Patch Density; LPI: Largest Patch Index; LSI: Landscape Shape Index; AWMSI: Area Weighted Mean Shape Index; ENN_MN: Mean Nearest Neighbor Distance; IJI: Interspersion and Juxtaposition Index; AI: Aggregation Index; HRULCI: Hydrological Response Unit Landscape Contrast Index.

**Table 4:** Calculation results of landscape pattern index in catchment area.

Year	NP	PD	LPI	LSI	AWMSI	ENN_MN	IJI	AI	HRULCI
2005	164,581	25.412	26.505	275.122	76.690	111.667	48.750	80.988	0.562
2010	17,115	2.623	31.433	81.770	22.736	291.034	66.903	94.220	0.653
2014	43,550	6.560	76.846	88.888	52.207	179.142	71.836	93.621	0.520

**Table 5:** The linear model general situation of pH.

pH Model	R	R^2^	Standard Estimation Error	Sig
pH-100	0.934 ^a^	0.872	0.109	0.271
pH-500	0.823 ^a^	0.677	0.172	0.702
pH-1000	0.818 ^a^	0.670	0.174	0.716
pH-2000	0.827 ^a^	0.685	0.170	0.690
pH-catchment	0.863 ^a^	0.744	0.153	0.576

^a^: predictive variables.

**Table 6:** The model significance table of pH.

Landscape Metrics	NP	PD	LPI	LSI	AWMSI	ENN_MN	IJI	AI	HRULCI
Significance	0.048	0.219	0.109	0.05	0.246	0.479	0.888	0.273	0.668

**Table 7:** The linear model general situation of DO.

DO Model	R	R^2^	Standard Estimation Error	Sig
DO-100	0.716 ^a^	0.513	1.117	0.902
DO-500	0.853 ^a^	0.728	0.835	0.609
DO-1000	0.904 ^a^	0.817	0.684	0.409
DO-2000	0.858 ^a^	0.736	0.822	0.593
DO-Catchment	0.885 ^a^	0.784	0.744	0.488

^a^: predictive variables.

**Table 8:** The linear model general situation of COD_Mn_.

COD_Mn_ Model	R	R^2^	Standard Estimation Error	Sig
COD_Mn_-100	0.935 ^a^	0.875	0.363	0.262
COD_Mn_-500	0.938 ^a^	0.880	0.356	0.250
COD_Mn_-1000	0.958 ^a^	0.918	0.294	0.153
COD_Mn_-2000	0.952 ^a^	0.907	0.314	0.181
COD_M__n_-catchment	0.987 ^a^	0.975	0.162	0.029

^a^: predictive variables.

**Table 9:** The model significance table of COD_Mn_.

Coefficients	Constant	NP	PD	LPI	LSI	AWMSI	ENN_MN	IJI	AI	HRULCI
Unstandardized Coefficients	−150.52	−7 × 10^−6^	0.031	−0.009	−0.01	0.024	−0.017	−0.06	0.207	110.483
Standard Estimation error	160.39	0	0.045	0.016	0.007	0.019	0.004	0.038	0.166	30.966
Significance	0.414	0.892	0.541	0.606	0.256	0.286	0.024	0.201	0.301	0.063

**Table 10:** The linear model general situation of TP.

TP Model	R	R^2^	Standard Estimation Error	Sig
TP-100	0.994 ^a^	0.988	0.032	0.010
TP-500	0.998 ^a^	0.996	0.017	0.002
TP-1000	0.993 ^a^	0.985	0.035	0.014
TP-2000	0.993 ^a^	0.987	0.033	0.012
TP-catchment	0.874 ^a^	0.764	0.139	0.532

^a^: predictive variables.

**Table 11:** The model significance table of TP.

Coefficients	Constant	NP	PD	LPI	LSI	AWMSI	ENN_MN	IJI	AI	HRULCI
Unstandardized Coefficients	−0.848	−5 × 10^−6^	0.01	0.02	0.002	−0.043	0.001	0.001	0.008	−0.213
Standard estimation	10.4	0	0.002	0.007	0.004	0.022	0	0.002	0.009	0.312
Significance	0.588	0.602	0.022	0.061	0.654	0.143	0.252	0.741	0.422	0.543

**Table 12:** The linear model general situation of NH_3_-N.

NH_3_-N Model	R	R^2^	Standard Estimation Error	Sig
NH_3_-N-100	0.960 ^a^	0.921	0.190	0.146
NH_3_-N-500	0.974 ^a^	0.950	0.151	0.079
NH_3_-N-1000	0.944 ^a^	0.890	0.224	0.223
NH_3_-N-2000	0.976 ^a^	0.953	0.147	0.072
NH_3_N-catchment	0.790 ^a^	0.624	0.414	0.784

^a^: predictive variables.

**Table 13:** The model significance table of NH_3_-N.

Coefficients	Constant	NP	PD	LPI	LSI	AWMSI	ENN_MN	IJI	AI	HRULCI
Unstandardized Coefficients	0.718	−3 × 10^−4^	0.051	0.03	0.022	−0.243	−0.003	−0.04	0.067	−40.157
Standard estimation	100.998	0	0.036	0.008	0.013	0.053	0.002	0.014	0.113	10.647
Significance	0.952	0.147	0.253	0.032	0.19	0.02	0.327	0.063	0.596	0.086

**Table 14:** The linear model general situation of TN.

TN Model	R	R^2^	Standard Estimation Error	Sig
TN-100	0.825 ^a^	0.680	20.667	0.698
TN-500	0.808 ^a^	0.652	20.780	0.743
TN-1000	0.849 ^a^	0.720	20.492	0.624
TN-2000	0.941 ^a^	0.885	10.597	0.236
TN-catchment	0.948 ^a^	0.900	10.493	0.199

^a^: predictive variables.

**Table 15:** The verification table of simulated water quality data.

Monitor Site-Water Quality Index	Simulation Data	Monitoring Data Range
Huaan-COD_Mn_	2.8	1.33–3.01
Huaan-TP	0.07	0.037–0.356
Huaan-NH_3_-N	0.573	0.017–1.56
Xiamen-COD_Mn_	1.94	2.62–5.02
Xiamen-TP	0.08	0.029–0.118
Xiamen-NH_3_-N	0.01	0.19–0.508

## References

[1] Xia L.L., Liu R.Z., Zao Y.W. (2012). Correlation analysis of landscape pattern and water quality in Baiyangdian Watershed. Procedia Environ. Sci..

[2] Qiu Z., Walter M.T., Hall C. (2007). Managing variable source pollution in agricultural watersheds. J. Soil Water Conserv..

[3] Diebel M.W., Maxted J.T., Robertson D.M., Han S., Vander Zanden M.J. (2009). Landscape planning for agricultural nonpoint source pollution reduction III: Assessing phosphorus and sediment reduction potential. Environ. Manag..

[4] Xin Z., Jintian C., Yuqi L., Lei W. (2017). Geo-cognitive computing method for identifying “source-sink” landscape patterns of river basin non-point source pollution. Int. J. Agric. Biol. Eng..

[5] Deng J.S., Wang K., Hong Y., Qi J.G. (2009). Spatio-temporal dynamics and evolution of land use change and landscape pattern in response to rapid urbanization. Landsc. Urban Plan..

[6] Jha M.K., Chowdhury A., Chowdary V.M., Peiffer S. (2007). Groundwater management and development by integrated remote sensing and geographic information systems: Prospects and constraints. Water Resour. Manag..

[7] Varanka S., Hjort J., Luoto M. (2015). Geomorphological factors predict water quality in boreal rivers. Earth Surf. Process. Landf..

[8] Ouyang W., Skidmore A.K., Toxopeus A.G., Hao F. (2010). Long-term vegetation landscape pattern with non-point source nutrient pollution in upper stream of Yellow River basin. J. Hydrol..

[9] Li H., Liu L., Ji X. (2015). Modeling the relationship between landscape characteristics and water quality in a typical highly intensive agricultural small watershed, Dongting Lake Basin, South Central China. Environ. Monit. Assess..

[10] Huang Z., Han L., Zeng L., Xiao W., Tian Y. (2016). Effects of land use patterns on stream water quality: A case study of a small-scale watershed in the Three Gorges Reservoir Area, China. Environ. Sci. Pollut. Res..

[11] Shen Z., Hou X., Li W., Aini G. (2014). Relating landscape characteristics to non-point source pollution in a typical urbanized watershed in the municipality of Beijing. Landsc. Urban Plan..

[12] Li Y., Li Y., Qureshi S., Kappas M., Hubacek K. (2015). On the relationship between landscape ecological patterns and water quality across gradient zones of rapid urbanization in Coastal China. Ecol. Model..

[13] Gonzales-Inca C.A., Kalliola R., Kirkkala T., Lepistö A. (2015). Multiscale landscape pattern affecting on stream water quality in agricultural watershed, SW Finland. Water Resour. Manag..

[14] De Oliveira L.M., Maillard P., Pinto E.J.D.A. (2017). Application of a land cover pollution index to model non-point pollution sources in a Brazilian watershed. Catena.

[15] Xiao R., Jiang D., Christakos G., Fei X., Wu J. (2016). Soil landscape pattern changes in response to rural anthropogenic activity across Tiaoxi watershed, China. PLoS ONE.

[16] Su S., Ma X., Xiao R. (2014). Agricultural landscape pattern changes in response to urbanization at ecoregional scale. Ecol. Indic..

[17] Shi P., Zhang Y., Li Z., Li P., Xu G. (2017). Influence of land use and land cover patterns on seasonal water quality at multi-spatial scales. Catena.

[18] Lv H., Cai J., Xu Y. Relationship between landscape pattern and water quality in north Jiangsu plain river network region, China. Proceedings of the International Conference on Geoinformatics.

[19] Xiao R., Wang G., Zhang Q., Zhang Z. (2016). Multi-scale analysis of relationship between landscape pattern and urban river water quality in different seasons. Sci. Rep..

[20] Yuqi L. (2018). A Study on the Relationships between Landscape Patterns and Water Quality across Multiple-Scales in Basin Based on Remote Sensing Technology. Master’s Thesis.

[21] Huang J.L., Li Q.S., Hong H.S., Lin J., Qu M.C. (2011). Preliminary study on linking land use & landscape pattern and water quality in the Jiulong River watershed. Environ. Sci..

[22] Parent J.R., Volin J.C. (2016). Validating landsat-based landscape metrics with fine-grained land cover data. Ecol. Indic..

[23] Shen Z., Hou X., Li W., Aini G., Chen L., Gong Y. (2015). Impact of landscape pattern at multiple spatial scales on water quality: A case study in a typical urbanised watershed in China. Ecol. Indic..

[24] Sun Y., Guo Q., Jian L., Wang R. (2014). Scale effects on spatially varying relationships between urban landscape patterns and water quality. Environ. Manag..

[25] McGarigal K., Marks B.J. Fragstats: Spatial Pattern Analysis Program for Quantifying Landscape Structure. https://www.fs.usda.gov/treesearch/pubs/3064.

[26] Herzog F., Lausch A., Muller E., Thulke H.H., Steinhardt U., Lehmann S. (2001). Landscape metrics for assessment of landscape destruction and rehabilitation. Environ. Manag..

[27] Uuemaa E., Roosaare J., Mander Ü. (2005). Scale dependence of landscape metrics and their indicatory value for nutrient and organic matter losses from catchments. Ecol. Indic..

[28] Amiri B.J., Nakane K. (2009). Modeling the linkage between river water quality and landscape metrics in the Chugoku District of Japan. Water Resour. Manag..

[29] Li S., Gu S., Liu W., Han H., Zhang Q. (2008). Water quality in relation to land use and land cover in the upper Han River Basin, China. Catena.

